# An Adaptive Impedance Matching Network with Closed Loop Control Algorithm for Inductive Wireless Power Transfer

**DOI:** 10.3390/s17081759

**Published:** 2017-08-01

**Authors:** Zhidong Miao, Dake Liu, Chen Gong

**Affiliations:** Institute of Application Specific Instruction-Set Processor, Beijing Institute of Technology, 5 South Zhongguancun Street, Haidian District, Beijing 100081, China; zhidongmiao@bit.edu.cn (Z.M.); gongchen@bit.edu.cn (C.G.)

**Keywords:** inductive wireless power transfer (IWPT), efficiency, output power, impedance matching network (IMN), adaptive IMN control

## Abstract

For an inductive wireless power transfer (IWPT) system, maintaining a reasonable power transfer efficiency and a stable output power are two most challenging design issues, especially when coil distance varies. To solve these issues, this paper presents a novel adaptive impedance matching network (IMN) for IWPT system. In our adaptive IMN IWPT system, the IMN is automatically reconfigured to keep matching with the coils and to adjust the output power adapting to coil distance variation. A closed loop control algorithm is used to change the capacitors continually, which can compensate mismatches and adjust output power simultaneously. The proposed adaptive IMN IWPT system is working at 125 kHz for 2 W power delivered to load. Comparing with the series resonant IWPT system and fixed IMN IWPT system, the power transfer efficiency of our system increases up to 31.79% and 60% when the coupling coefficient varies in a large range from 0.05 to 0.8 for 2 W output power.

## 1. Introduction

**Figure 1:**
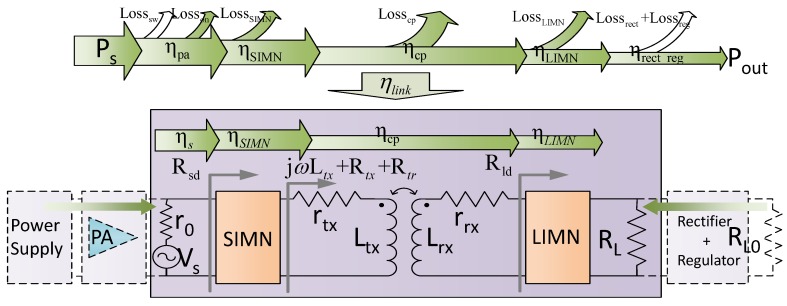
A conventional inductive wireless power transfer (IWPT) system structure. $\eta_{pa}$, the efficiency of power amplifier (PA); $\eta_{SIMN}$, the efficiency of SIMN; $\eta_{cp}$, the efficiency of the coupled coils; $\eta_{LIMN}$, the efficiency of LIMN; $\eta_{\eta_{rect\_reg}}$, the efficiency of the rectifier and the regulator; $\eta_{link}$, the efficiency of simplified IWPT system model, which is the focus of this paper; $Loss_{sw}$, the switch loss of PA; $Loss_{on}$, the switch-on resistance loss of PA; $Loss_{SIMN}$, the SIMN loss; $Loss_{cp}$, the coupled coil loss; $Loss_{LIMN}$, the LIMN loss; $Loss_{rect}$, the rectifier loss; $Loss_{reg}$, the regulator loss; $R_{sd}$ the equivalent load of the source; $R_{ld}$, the equivalent load resistance at the input port of LIMN; $R_{tr}$, the reflected resistance from load side into the source side.

IWPT technology can provide power without physical connections. Due to this unique feature, IWPT technology has attracted much attention in applications such as battery charging, portable devices, RFID, and medical devices [1,2,3,4,5]. Since the coupling between the inductors in IWPT systems is usually weak, it is a great challenge to obtain a high efficiency. The efficiency distribution for a conventional IWPT system is shown in Figure 1. In order to obtain a high efficiency, many optimization efforts have been made by researchers on every step of transferring and coupling, such as the power amplifier [6,7], the coupling coil geometry [4,8,9], the load [10], and the impedance matching networks [11,12,13,14,15,16]. Most of these researches are discussed under fixed relative position of the coils, which means that the coupling is fixed. However, in most applications, the distance is varied which leads to two problems: one is the efficiency degradation, another is the load power variation on load side.

In order to dynamically adapt IWPT to more application scenarios, these problems must be solved and several technologies have been proposed in the literature, including the voltage adjustment, the PWM duty cycle adjustment, the frequency tracking, the load resistance adjustment and the capacitor or inductor adjustment, etc. The supply voltage adjustment and the PWM duty cycle adjustment are two methods used in Qi standard to control the output power of the power amplifier (PA) [17]. However these two methods can only control the power delivered to load, and are helpless to minimize the power efficiency degradation when the coupling coil distance is large. Frequency tracking is a concise method to maintain high efficiency by tuning the system adaptively. However, a relatively large allowed frequency spectrum band is needed. Since frequency spectrum is limited resource, large spectrum band requirement limits its application [18,19]. Garnica et al. [20] and Zargham et al. [11] demonstrated that an optimum load resistance exists, with which the coupling efficiency ($\eta_{cp}$ in Figure 1) can be maximized. According to this theory, the load resistance is adjusted in [21,22,23] to adapt to distance variation. These methods achieve a good performance in power efficiency. However, the output power is varied with the efficiency, which is not controlled independently.

Another promising solution is reconfiguring the capacitors in the IMN [12,13,24,25]. IMN can not only compensate the mismatch of the imaginary part of the load, but also transform the real part of the load for the optimum matching. For the IMN at load side (LIMN), this resistance transformation can transform the load into the optimal value we mentioned above; and for the IMN at source side (SIMN), this resistance transformation can modify the PA output power by modifying the load impedance of PA. When the distance varies, by re-matching the IMN, the power transfer efficiency could be enhanced since the power reflection caused by mismatch is diminished, and the power delivered to load could also be adjusted. In [12,13], a look-up table is used to record the optimal capacitor values in IMN for various coil distances. Some search algorithms are also provided in [13] to search the optimal capacitors when distance varies. These scenarios only optimize the IMN at several fixed points, and the control process does not form a closed feedback loop, which limit their performances on efficiency and stability. In [24], series resonant capacitors are used to tune the coils. The voltage-current phase difference for the PA output signal are monitored to adjust the resonant capacitors automatically, by which the phase difference keeps zero. This system is a closed loop control system, and the efficiency degradation caused by distance variation can be partly compensated by compensating the mismatch. However their output power is not controllable. In [25], The matching capacitor is calculated by measuring the S parameter of the system. Although this method could achieve a precise adjustment, the S parameter is measured by VNA which is difficult to implement.

**Figure 2:**
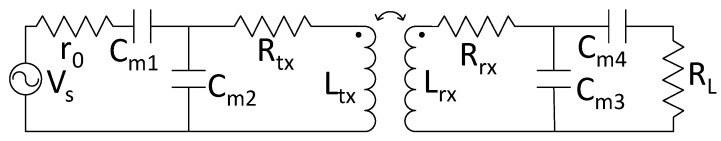
The Γ type IMNs used in our adaptive IMN IWPT system. $C_{m1}$ and $C_{m2}$ make up SIMN, and $C_{m3}$ and $C_{m4}$ make up LIMN.

Since adjusting IMN has many advantages as we mentioned above, and the existing scenarios still have some weaknesses. We proposed a new adaptive IMN IWPT system in this paper. The Γ type IMNs shown in Figure 2 are used in our adaptive IMN IWPT system. By analysing the matching process of Γ type SIMN, we find that the two capacitors in SIMN show two independent functions: $C_{m1}$ is used to compensate the mismatch of the imaginary part of SIMN input impedance, and $C_{m2}$ is used to adjust the real part of the SIMN input impedance. Thus, by controlling these two components simultaneously, both of the mismatch compensation of imaginary part and the output power control can be accomplished. Our proposed adaptive IMN IWPT system is designed based on the review above, which could cover a large distance variation with high power efficiency and stable output power. Furthermore, our adaptive IMN IWPT system is a closed loop feedback system, which could perform a high robust. The experiment results show that our IWPT system can work under the coupling coefficient ranging from 0.05 up to 0.8 with a high efficiency up to 76%. Comparing with the series resonant IWPT system and fixed IMN IWPT system, the power transfer efficiency of our system increases up to 31.79% and 60%.

The rest of the paper is organized as follows. Section 2 analyses the optimal IMN design for maximum power efficiency under fixed distance. Section 3 analysis the matching process of IMN and proposes our adaptive IMN solution to resist distance variation. Section 4 presents the implementation details of our adaptive IMN IWPT system. The experiment verification of the performance is shown in Section 5. Finally, conclusions are drawn in Section 6.

## 2. IMN Design Analysis under Fixed Coupling Coefficient

In this section, the IMN optimization under fixed distance (means fixed coupling coefficient) is discussed. A conventional IWPT system is shown in Figure 1. In order to simplify the analysis, the power amplifier (PA) is simplified as a sinusoidal voltage source $V_{s}$ in series with a resistor, and the rectifier, the regulator and the load are simplified as a resistance load. An IWPT system specified in Table 1 is used in our discussion. The coils we used are manufactured under Qi standard by Würth [26]. These coils are both spiral winded with copper wires and adhered to a shielding plate made by soft-magnetic material. Although our analysis is based on this specific application, the analysis and method presented in this paper are applicable for any other IWPT applications.

The SIMN or LIMN can be a resonant capacitor or other more complex IMN structures, such as Γ type capacitor matching network. At the load side, the LIMN transforms the load $R_{L}$ into $-j\omega L_{rx}+R_{ld}$, in which, $-j\omega L_{rx}$ compensates the inductance of the receiving coil $j\omega L_{rx}$ and the value of $R_{ld}$ is decided by the designer. At the source side, the SIMN transforms the load $R_{tx}+R_{tr}+j\omega L_{tx}$ into $R_{sd}$, by which the inductance of the transmitting coil $j\omega L_{tx}$ has been compensated, and $R_{sd}$ is also need to be decided by the designer. $R_{tr}$ represents the reflected resistance from the receiving side. From the analysis we can see that when the system is compensated, the resistance transformations of the IMNs ($R_{L}$ to $R_{ld}$ and $R_{tx}+R_{tr}$ to $R_{sd}$) need to be decided by the designers, which are opportunities to enhance the efficiency and adjust the power delivered to load.

For a conventional IWPT system, the power transfer efficiency can be represented as

(1) $$\begin{array}{c} \begin{array}{c} \eta_{link}=\eta_{s}\eta_{SIMN}\eta_{cp}\eta_{LIMN} \end{array} \end{array}$$

The IMNs are usually configured by capacitors whose loss are usually negligible. So the efficiency can be simplified as

(2) $$\begin{array}{c} \begin{array}{c} \eta_{link}=\eta_{s}\eta_{cp}=\frac{R_{sd}}{r_{0}+R_{sd}}\frac{\frac{\omega^{2}k^{2}L_{tx}L_{rx}}{R_{rx}+R_{ld}}}{R_{tx}+\frac{\omega^{2}k^{2}L_{tx}L_{rx}}{R_{rx}+R_{ld}}}\frac{R_{ld}}{R_{ld}+R_{rx}} \end{array} \end{array}$$

In this equation, the frequency, source resistance, coil inductances and resistances are decided by the application specification, the coupling coefficient *k* is varied when the relative distance is varied, the two controllable parameters we can use to optimize the system is $R_{ld}$ and $R_{sd}$, which just meet with our analysis above. The adjustment of the $R_{ld}$ and $R_{sd}$ are accomplished by configuring LIMN and SIMN.

For LIMN, when $R_{ld}$ equals to the following equation

(3) $$\begin{array}{c} \begin{array}{c} R_{ld\_opt}=R_{rx}\sqrt{1+\frac{\omega^{2}M_{rx-tx}^{2}}{R_{tx}R_{rx}}} \end{array} \end{array}$$

the maximum $\eta_{cp}$ value can be derived from [27]

(4) $$\begin{array}{c} \begin{array}{c} \eta_{cp\_opt}=\frac{\frac{\omega^{2}k^{2}L_{tx}L_{rx}}{R_{tx}R_{rx}}}{{\left(1+\sqrt{1+\frac{\omega^{2}k^{2}L_{tx}L_{rx}}{R_{tx}R_{rx}}}\right)}^{2}} \end{array} \end{array}$$

The SIMN can also influence $\eta_{link}$ by altering $R_{ld}$, which is the main consideration of this paper. From the PA point of view, $R_{ld}$ is the load. So the adjustment of $R_{ld}$ influences the PA output power as well as the PA efficiency.

The power delivered to load of the system can be calculated by

(5) $$\begin{array}{c} \begin{array}{c} P_{out}=\frac{V_{s}^{2}R_{sd}}{2{\left(r_{0}+R_{sd}\right)}^{2}}\eta_{cp} \end{array} \end{array}$$

From Equations (2) and (5), we can see that, by letting $P_{out}$ equal to load required power level $P_{req}$, the power transfer efficiency is maximized. So, the expression for optimal $R_{sd\_opt}$ is derived as

(6) $$\begin{array}{c} \begin{array}{c} R_{sd\_opt}=\frac{V_{s}^{2}}{4P_{req}}\frac{\frac{\omega^{2}k^{2}L_{tx}L_{rx}}{R_{tx}R_{rx}}}{{\left(1+\sqrt{1+\frac{\omega^{2}k^{2}L_{tx}L_{rx}}{R_{tx}R_{rx}}}\right)}^{2}}-r_{0}+\\ \;\frac{V_{s}}{4P_{req}}\sqrt{V_{s}^{2}{\left[\frac{\frac{\omega^{2}k^{2}L_{tx}L_{rx}}{R_{tx}R_{rx}}}{{\left(1+\sqrt{1+\frac{\omega^{2}k^{2}L_{tx}L_{rx}}{R_{tx}R_{rx}}}\right)}^{2}}\right]}^{2}-8r_{0}P_{req}\frac{\frac{\omega^{2}k^{2}L_{tx}L_{rx}}{R_{tx}R_{rx}}}{{\left(1+\sqrt{1+\frac{\omega^{2}k^{2}L_{tx}L_{rx}}{R_{tx}R_{rx}}}\right)}^{2}}} \end{array} \end{array}$$

The optimal $R_{ld}$ and $R_{sd}$ is implemented by LIMN and SIMN separately. The values for IMN components can be determined by smith chart tool in advanced design system (ADS).

## 3. Adaptive IMN Algorithm for Distance Variation

**Figure 3:**
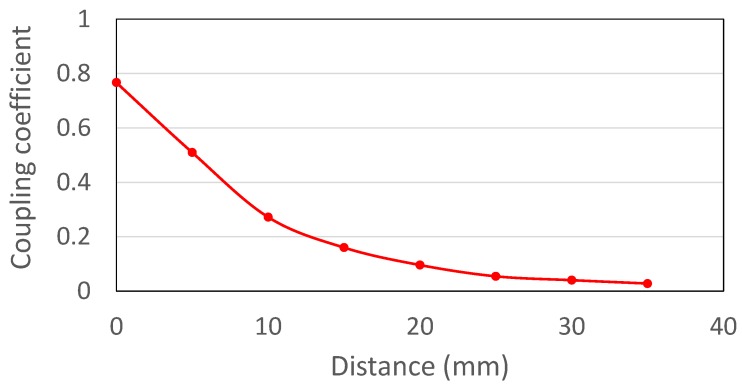
The measured coupling coefficient (*k*) variation when two coils are aligned and the distance varies from 0 to 35 mm.

The analysis in last section provides the optimal $R_{ld}$ and $R_{sd}$ for maximizing the power transfer efficiency under the fixed coupling coefficient. However, in real applications, the distance is usually uncertain, by which the coupling coefficient varies. The distance and relative position dependent coefficient variation is measured and shown in Figure 3. The larger the distance, the lower the coupling coefficient. A robust IWPT design need to cover the distance variation as large as possible.

**Figure 4:**
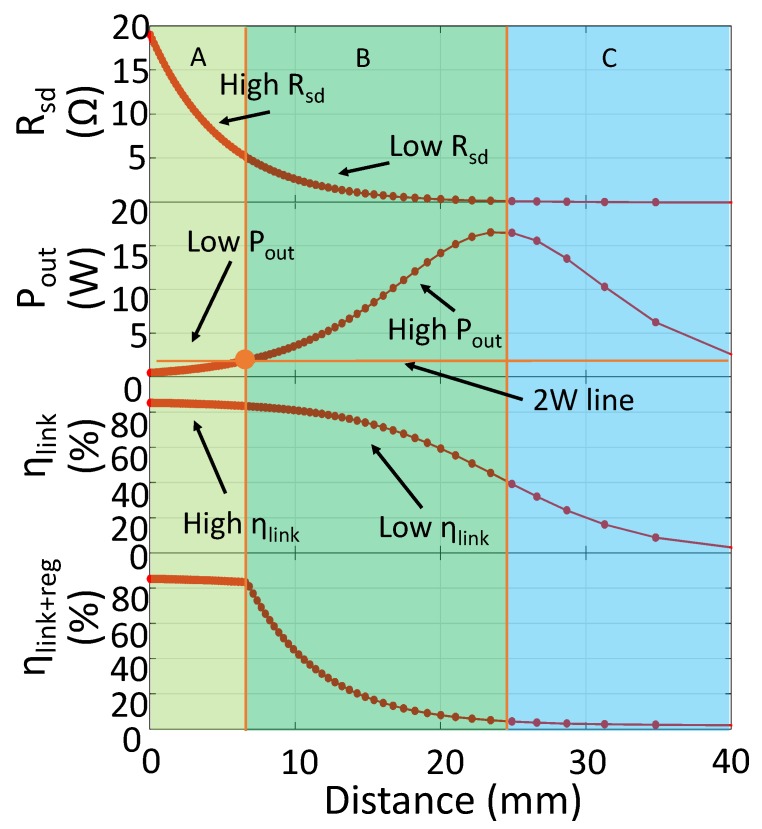
The IWPT system performance when IMN is a series resonant capacitor. $R_{sd}=R_{tx}+R_{tr}$. The supply voltage is constant as $V_{s}=5$ V. $\eta_{link+reg}$ represents the power efficiency assuming that the redundant power delivered to load is wasted on regulator. When system is in region B and C in the figure, the output power is higher than the load needed power level. This redundant power would be wasted at the load side which decreases the efficiency. In region A, there is no redundant power loss at load side, but the output power is less than needed.

To evaluate the performance drop by distance variation, we set LIMN according to Equation (3) and k=0.1 (the detail analysis for LIMN selection is presented in Section 4.1), and we also set the SIMN as the series resonant capacitor ($C_{m2}=0$). Series resonant capacitor could compensate the coil inductance , but it can not change the real part of SIMN input impedance. The system is powered by a constant voltage supply with $V_{s}=5$ V. The load power requirement is 2 W. In this series resonant SIMN IWPT system, $R_{sd}$ equals to the resistance across the transmitting coil $R_{tr}+R_{tx}$. The system performance is shown in Figure 4. We can see that the plot can be divided into three region. In region A, $R_{sd}$ is large, which leads to high efficiency. However large $R_{sd}$ decreases the PA output power, by which the power delivered to load is low. The boundary between region B and A is the point at which the output power level just equals to the load needed power level. At region B, when *k* decreases, the output power increases but the efficiency decreases inversely. If the redundant power delivered to load is not used, this power will be wasted in the regulator, thus the efficiency decreases further. The boundary of region C and B is through the point at which $R_{sd}=r_{0}$. In region C, the $\eta_{s}$ is less than 50%, which is too low to accept. From the coupling point of view, region A can be seen as the strong coupling region, and region B and C is the week coupling region. In this paper, we focus on providing an adaptive IMN IWPT system covering region A and B.

For an adaptive IWPT system, there are two issues must to be addressed. The first one is the compensation of the imaginary part of the SIMN input impedance. When the coil distance is varied, the SIMN input impedance will be varied. We must ensure that the imaginary part of this impedance is compensated so that there is no power reflection. The system efficiency will also be enhanced by this step. The compensation of the imaginary part of SIMN input impedance can be accomplished by configuring SIMN.

The second one is the output power adjustment. To maximize the power efficiency, we should keep the output power equal to the load power requirement. The output power is not the larger the better, because the redundant power will be wasted. There are two methods to control the output power: one is to adjust the supply voltage, the other one is to adjust $R_{sd}$. $R_{sd}$ adjustment can be accomplished by adjusting SIMN.

At region A, the power transfer efficiency is high. However, since the $R_{sd}$ is too large, the the volume of the output power is less than load requirement. Thus to maintain the high efficiency, the output power should be controlled by adjusting the supply voltage. In this region, to minimize $R_{sd}$, $C_{m2}$ is reduced to zero so that SIMN is series resonant circuit. At region B, the situation is just on the contrary. The $R_{sd}$ is low, which decreases the efficiency and makes the output power level higher than needed. For this region, adjusting $R_{sd}$ is a better choice. By increasing $R_{sd}$, not only the output power can be decreased, but also the efficiency can be increased as well. This is because high $R_{sd}$ means that PA drives a high load impedance which decreases the PA loss.

Our design strategy for region A and B are summarized as:

1. For region A, $C_{m2}=0$, SIMN is series resonant with $L_{tx}$, $V_{s}$ is adjusted to adapt to load needed power level.
2. For region B, $V_{s}$ is constant, SIMN is reconfigured to let $R_{sd}=R_{sd\_opt}$ as Equation (6), by which the output power also adapt to load needed power level.

In this design strategy, the SIMN is reconfigured for both the compensation for the imaginary part of SIMN input impedance and the $R_{sd}$ adjustment. Thus, a control system and algorithm is needed to reconfigure the SIMN in Figure 2.

To address this, the matching process of the Γ type SIMN is analysed and a SIMN control strategy is proposed. Since for region A, SIMN is series resonant which is a special case for $C_{m2}=0$, the following analysis is mainly for region B where $C_{m2}\neq 0$. However, finally, we can find that region A can also be involved into our IMN control strategy.

**Figure 5:**
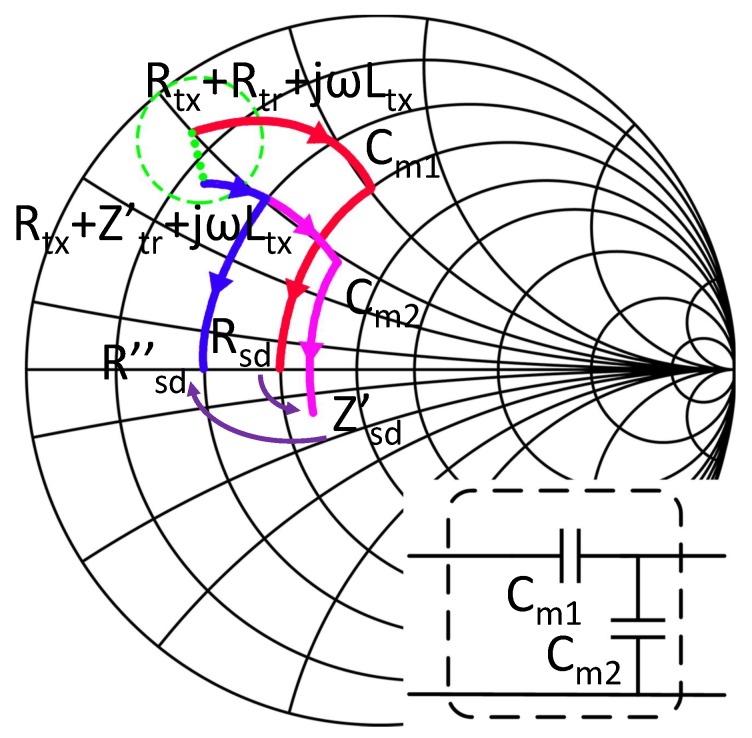
The mismatch and rematch process shown in smith chart. The red curve is the original state of the SIMN; the pink curve is the mismatch state when the coupling or load is varied and the SIMN is not adjusted; the blue curve is the matching state when the SIMN is adjusted according to the variation.

The mismatch and rematch process of SIMN is shown in Figure 5. When coupling coefficient is varied, the $R_{sd}$, and the reflected impedance $R_{tr}$ are varied to $Z_{sd}^{{}^{\prime }}$ and $Z_{tr}^{{}^{\prime }}$. Under this new coupling coefficient, the optimal $R_{sd}$ is also modified to $R_{sd}^{{}^{\prime \prime }}$. So the reconfiguring of the SIMN is to transform the input impedance of SIMN from $Z_{sd}^{{}^{\prime }}$ to $R_{sd}^{{}^{\prime \prime }}$. To achieve this rematch, the most direct method is deriving $R_{sd}^{{}^{\prime \prime }}$ from Equation (6) and then calculating the corresponding $C_{m1}$ and $C_{m2}$ by ADS. However, since it is hard to get the definite value of coupling coefficient, we cannot derive $R_{sd}^{{}^{\prime \prime }}$ from Equation (6) directly.

**Figure 6:**
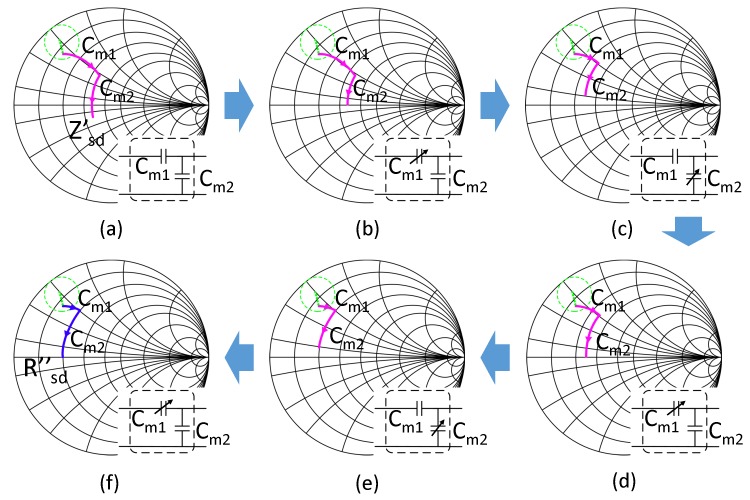
The IMN capacitor adjusting process to rematch the system from the pink curve in Figure 5 into the blue curve. (**a**) The mismatched IMN; (**b**) adjusting $C_{m1}$ to compensate the imaginary part of impedance; (**c**) adjusting $C_{m2}$ to match the real part of the impedance; (**d**) adjusting $C_{m1}$ to compensate the imaginary part of impedance; (**e**) adjusting $C_{m2}$ to match the real part of the impedance; (**f**) adjusting $C_{m1}$ to compensate the imaginary part of impedance.

Thus, a more practicable solution is needed. The optimal $R_{sd\_opt}$ from Equation (6) is derived by compensating the imaginary part of the $Z_{sd}^{{}^{\prime }}$ ($Im\{Z_{sd}^{{}^{\prime }}\}$) and equalling the output power to the load needed power level. We try to find another way to accomplish these targets. $Im\{Z_{sd}^{{}^{\prime }}\}$ is proportional to the voltage-current phases angle Δθ(V-I) at the SIMN input port, and $R_{sd}$ is proportional to the voltage across the load. If we can find a relationship among $Im\{Z_{sd}^{{}^{\prime }}\}$, $V_{RL}$ and the SIMN capacitor values, a closed loop control system can be established to rematch the system adaptively. Fortunately, one kind of this relationship is found by us. We found that $C_{m1}$ compensates the imaginary part of $Z_{sd}$, and $C_{m2}$ determines the real part of $Z_{sd}$. This means that $C_{m1}$ is proportional to Δθ(V-I) and $C_{m2}$ is proportional to $V_{RL}$. So we can make up a closed loop control system based on this. We also notice that when $C_{m2}$ changes, not only $Re\{Z_{sd}^{{}^{\prime }}\}$ changes, but also $Im\{Z_{sd}^{{}^{\prime }}\}$ changes. Thus, whenever we change $C_{m2}$, $Im\{Z_{sd}^{{}^{\prime }}\}$ is mismatch, and $C_{m1}$ should be changed accordingly. Thus, the adjustment of $C_{m1}$ and $C_{m2}$ should be a staggered and iterative process. An capacitor adjusting process based on the discussion above is shown in Figure 6, which transform $Z_{sd}^{{}^{\prime }}$ into $R_{sd}^{{}^{\prime \prime }}$.

All above discussion is for region B, which can also involve region A in. When the distance decreases, the reflected impedance $Z_{tr}$ increases. In order to ensure the power delivered to load, the optimal $R_{sd}$ is decreased. This means $R_{sd}$ approaches to the $Z_{tr}+R_{tx}$ and $C_{m2}$ approaches to zero when distance decreases. When $Z_{sd}=Z_{tr}+R_{tx}$, $C_{m2}$ equals to zero, the SIMN only compensates $Im\{Z_{sd}^{{}^{\prime }}\}$, and degrades into a series resonant capacitor. So, the system enters into region A. After the system entering region A, $C_{m2}=0$, $C_{m1}$ is adjusted to compensate $L_{tx}$. Since $R_{sd}$ is so large that the output power does not meet the load needed power level, and there is no margin for us to improve output power by adjusting $C_{m1}$. So, the supply voltage $V_{s}$ is adjusted accordingly.

**Figure 7:**
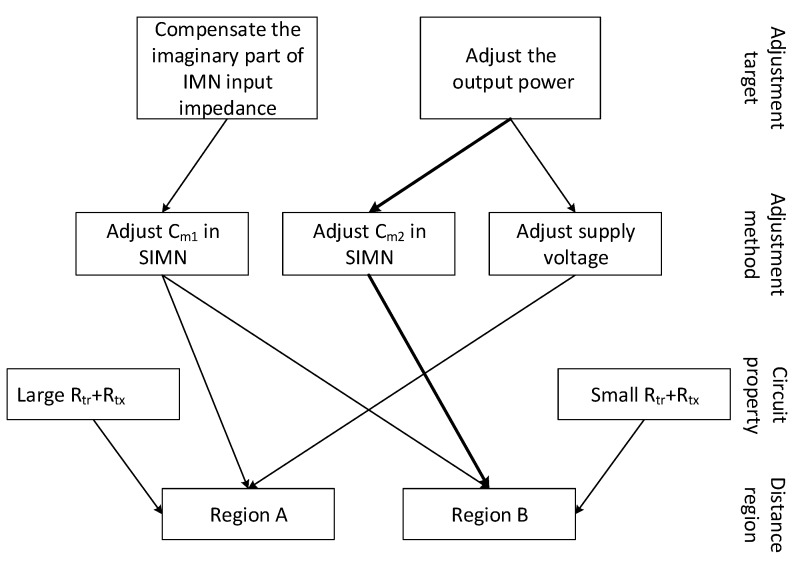
The relationship among the adjustment targets, the control methods, the SIMN impedance for region A and B. $R_{tx}+R_{tr}$ is the resistance across the transmitting coil, which equals to $R_{sd}$ when SIMN is series resonant as shown in Figure 4. For region A, $R_{tx}+R_{tr}$ is large, the output power is adjusted by controlling the supply voltage. For region B, $R_{tx}+R_{tr}$ is small, the output power is adjusted by controlling the $C_{m2}$ in SIMN. For both region A and B, the compensation of the imaginary part of SIMN input impedance is accomplished by adjusting $C_{m1}$ in SIMN.

The relationship among the adjustment targets, the control method and the SIMN impedance is shown in Figure 7. In addition, according to all above discussions, the control of supply voltage and SIMN capacitors for region A and B, can be integrated into one control algorithm as below:

1. Monitor the $Im\{Z_{sd}\}$, which is equal to the difference of the voltage and current phase angles Δθ(V-I).
2. If Δθ(V-I)>0, increase $C_{m1}$, otherwise decrease $C_{m1}$, until Δθ(V-I)=0.
3. When Δθ(V-I)=0, detect the voltage across load $V_{RL}$. If $V_{RL}-V_{RL\_req}>0$, increase $C_{m2}$, otherwise decrease $C_{m2}$, until $V_{RL}-V_{RL\_req}=0$.
4. When the $C_{m2}$ decreases to zero, this means the system goes into region A. Then, the SIMN is fixed and $V_{s}$ is adjusted as below: detecting $V_{RL}$, if $V_{RL}-V_{RL\_req}>0$, decrease $V_{s}$, otherwise increase $V_{s}$, until $V_{RL}-V_{RL\_req}=0$.

Based on the strategy above, a control algorithm for our adaptive IMN IWPT system is proposed as Algorithm 1. In this algorithm, $V_{RL}$ and $V_{RL\_req}$ are used to represent the output power level and load needed power level. To ensure the system stability, dead zones are added for θ(V,I) and $V_{RL}-V_{RL\_req}$. In our implementation, $\theta_{deadzore}$ and $V_{deadzone}$ are set as $5^{\circ }$ and 0.2 V. θ(V,I) is compensated with $\theta_{0}\left(V,I\right)$ considering the different signal delay, which is discussed in next section. $C_{step}$ and $V_{s\_step}$ are step size for capacitor and $V_{s}$ adjustments. $V_{s0}$ is the constant initial voltage for system working at region B. These parameters are discussed in next section.
**Algorithm 1:**
**Adaptive Control Algorithm** **Input**: $V_{RL}$, θ(V,I),$\theta_{0}\left(V,I\right)$, $V_{s0}$ **Output**: $V_{s}$, $C_{m1}$, $C_{m2}$

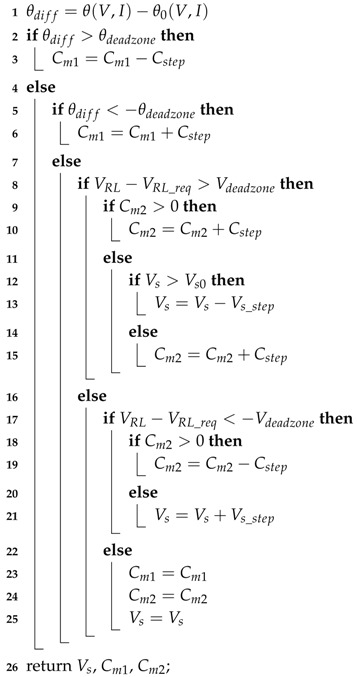



## 4. Implementation of the Proposed Adaptive IMN IWPT System

**Figure 8:**
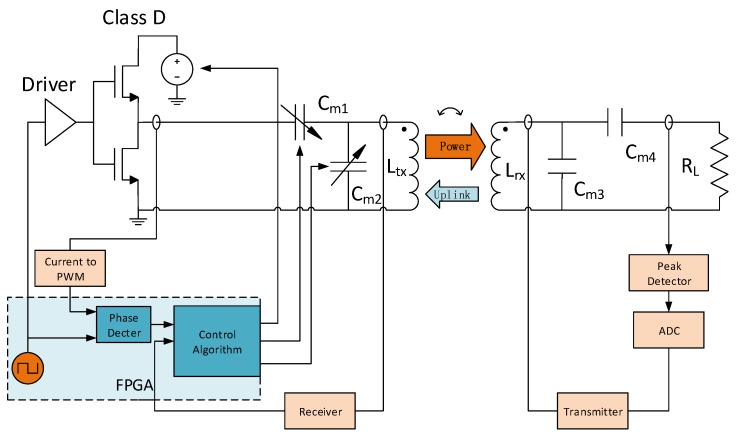
The system block diagram of our proposed adaptive IWPT system.

In this section, an adaptive IMN IWPT system according to the algorithm presented in the previous section is proposed which is shown in Figure 8. In this prototype demo, the IWPT system is driven by a class D PA; the IMNs are used to match with the coupled coils. In order to estimate the output power, the load voltage is measured by a peak detect circuit and an ADC, and the result is sent into the source side by a communication transceiver system. This transceiver system is discussed in detail in another paper [28] which will not be discussed in this paper. At the source side, in order to estimate the angle of $Z_{sd}$, the voltage-current phase difference (θ(V,I)) across the SIMN input port is measured. The measured output voltage and θ(V,I) are then used to adjust the SIMN capacitors and the $V_{s}$. The power supply of the PA is from a DC-DC converter LT1370, whose output voltage is controlled by an adjustable resistor at its feedback pin. A FPGA is used as the controller.

When the system power is on, FPGA generates a PWM signal to drive the PA. The PA output current is monitored by the module of Current to PWM and the result is sent into the FPGA. At load side, the peak voltage across the load is detected by peak detector module and sent to the FPGA by a wireless transceiver system [28]. In FPGA, the monitored current and peak voltage across load are used to evaluate θ(V,I) and $V_{RL}$. In addition, according to θ(V,I) and $V_{RL}$, our adaptive control algorithm is processed in FPGA, by which $C_{m1}$ and $C_{m2}$ are modified. When the control algorithm works continuously, the system can adapt to the variation of the coil distance automatically.

The design details of the circuit blocks in Figure 8 are discussed below.

### 4.1. Design of The LIMN and SIMN

LIMN and SIMN are two critical parts in our adaptive IMN IWPT system, which needs to be designed carefully.

**Figure 9:**
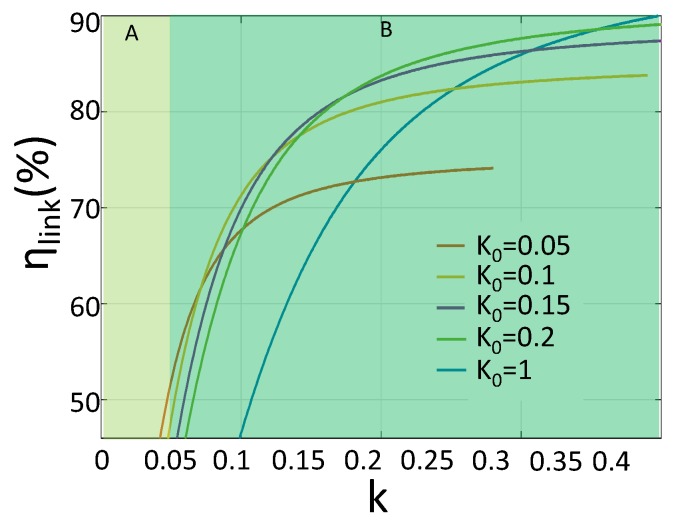
The IWPT system efficiency vs. *k* for different initial $k_{0}$. For $k_{0}=1$, the LIMN is in series resonant with $L_{rx}$.

For LIMN, its optimal value is related to coupling coefficient k as Equation (3). However, *k* is hard to detect, which make it impossible to adjust LIMN accordingly. So, in our system, LIMN is fixed, its capacitor values is determined under a certain coupling coefficient $k_{0}$. The chosen of $k_{0}$ will influence the system performance. Figure 9 shows the system performance under different $k_{0}$. $k_{0}=0.05$ could work in the most smallest *k*, but at high *k* region, the efficiency is significantly smaller than the others. $k_{0}=0.1$ could cover the second largest *k* region and the efficiency at high *k* region is also large enough. Thus, we choose $k_{0}=0.1$ as the initial *k* for LIMN. With this $k_{0}$, the value of the LIMN is: $C_{m3}=98$ nF, $C_{m4}=68$ nF. In addition, k=0.05 is the smallest coupling coefficient our system can cover.

**Figure 10:**
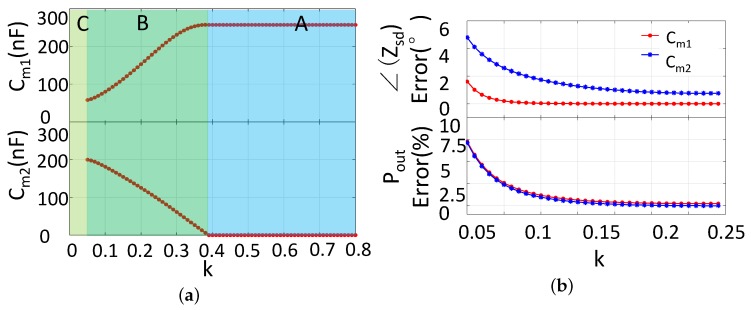
(**a**) The SIMN capacitor values for the coupling coefficient variation; (**b**)The phase error and output error caused by the inaccurate capacitor adjustment, assuming that the capacitor adjusting step is 1 nF.

SIMN is reconfigurable in our adaptive IMN IWPT system. The reconfiguration is achieved by capacitor array with relays. The capacitor value of $C_{m1}$ and $C_{m2}$ is controlled by switching the connected relay. The variation range and varying step for $C_{m1}$ and $C_{m2}$ should be determined. We calculated the capacitor values under different coupling coefficient, which is shown in Figure 10a. From the figure we can see that the variation range for $C_{m1}$ and $C_{m2}$ are 58–257 nF and 0–200 nF respectively. The capacitor value variations are continuous along with the coupling coefficient variation. For the capacitor array, the smaller is the capacitor varying step, the higher is the adjustment resolution. However, the small varying step leads to large capacitor array and complex driving circuit. By trading off, we choose 1 nF as the varying step for the demonstration demo. The $Z_{sd}$ deviation and the $P_{out}$ deviation is shown in Figure 10b when the capacitor has 1 nF step size. The largest deviation of the $Z_{sd}$ angle is less than $5^{\circ }$, and the largest deviation of the output power is less than 10%. This kind of deviation is small enough for the demo. The capacitor values in the array to cover $C_{m1}$ and $C_{m2}$ variations are listed in Table 2. For $C_{m1}$, 9 capacitor elements are used, and for $C_{m1}$, 8 capacitor elements are used. The capacitor array size is also affordable.

In our analysis, the Q loss of IMN is not considered to simplify the analysis. However, in real system implementation, the capacitor and relay should be chosen carefully. Otherwise, the Q loss of IMN may be unaffordable. In our system, negative-positive-zero (NPO) type capacitor with low parasitic resistance is used. The parasitic resistance is lower than 100 mΩ when capacitor range is from 1 nF to 200 nF. We use AGQ2104H as the relay in our system. This relay has a latch function, by which the relay consumes no power at steady state. The contact resistance for the relay switch is also lower than 100 mΩ.

### 4.2. Design of the Voltage-Current Phase Angle Detector

**Figure 11:**
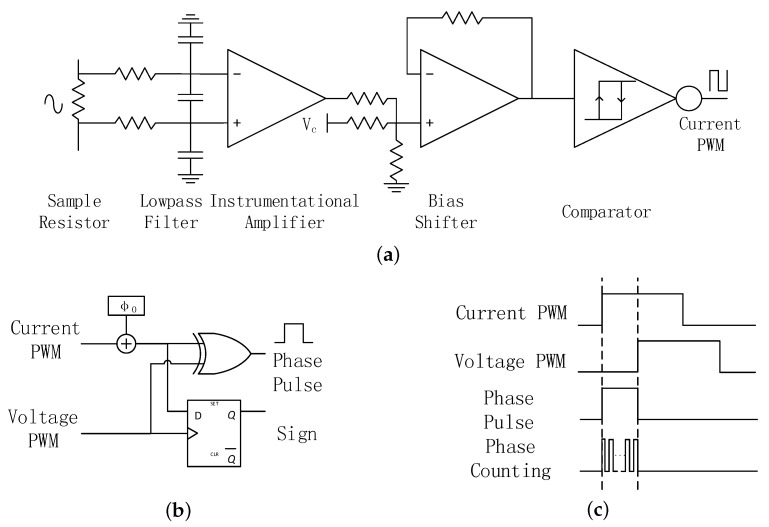
The voltage-current phase detector: (**a**) the current signal detector; (**b**) the voltage-current phase detect algorithm; (**c**) the PWM signals in the phase detect process.

**Figure 12:**
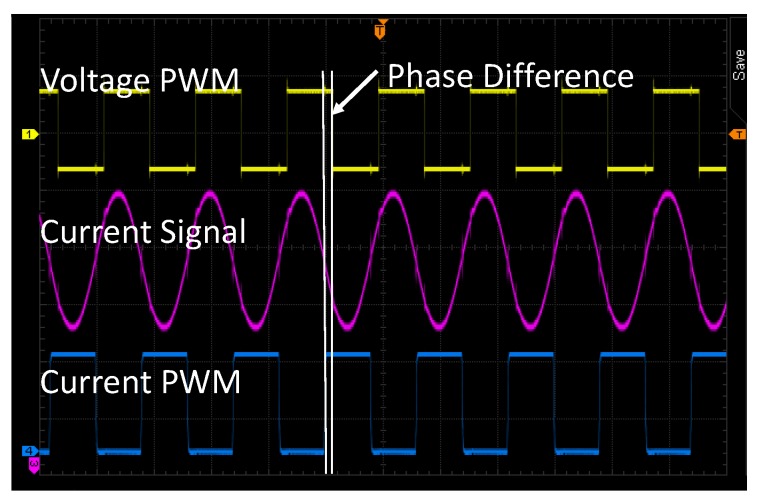
Tested voltage-current phase angle signal. The monitored sinusoidal current signal is transformed into PWM signal. The phase difference between voltage PWM and current PWM shown in the figure is measured by the FPGA logic.

Figure 11 shows the circuit implementation of the voltage-current phase detector. The PA drive signal, voltage PWM, can be used to represent voltage phase directly. The current phase signal needs to be captured from the input port of SIMN. The current phase extraction circuit is shown in Figure 11a. Firstly, the current signal is sampled by a resistor. Secondly, the sampled signal passes a low-pass filter to attenuate the noise. Thirdly, an instrumental amplifier is used to amplify the signal. Fourthly, the signal bias is shifted to fit with the comparator threshold voltage. In addition, fifthly, a comparator converts the current signal into a current PWM signal which represents the current phase. The tested voltage PWM and current PWM from this system is shown in Figure 12.

With the voltage PWM and current PWM, a digital algorithm is applied in FPGA to get θ(V,I), which is shown in Figure 11b,c. Since the current PWM is captured from the input port of SIMN, the detect circuit will introduce in a signal delay, which need to be compensated by a initial phase $\Phi_{0}$. The sign of θ(V,I) is derived from a D flip-flop, and the magnitude of θ(V, I) is proportional to the PWM width of the XOR logic output, which is measured by a pulse width counter. The deviation of the $Z_{sd}$ angle caused by step quantized capacitor in SIMN is less than $5^{\circ }$. For the pulse width counter, the accuracy must be less than $5^{\circ }$ so that the pulse width counter introduced deviation is negligible for the demo. Thus, the counting frequency is set to 360 times of the working frequency (125 kHz, the frequency for current PWM and voltage PWM) which is 45 MHz. This counting frequency can provide $1^{\circ }$ accuracy for the counter.

Before running the system, the initial value of $\Phi_{0}$ need to be set to compensate the path delay. We set $C_{m2}=0$ firstly. Then, we adjust $C_{m1}$ until the voltage across load is maximized. Under this condition, the SIMN is in series resonant with $L_{tx}$ and the θ(V,I) should be zero. We thus set the counted θ(V,I) as $\Phi_{0}$.

### 4.3. Design of the Load Received Power Detector

**Figure 13:**
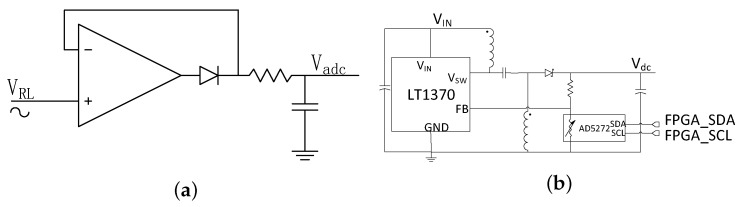
(**a**) The voltage peak detect circuit; (**b**) The supply voltage adjustment circuit.

The power level delivered to load is evaluated by measuring the peak voltage across the load. A peak detect circuit shown in Figure 13a is used to capture the peak voltage. Then an ADC is used to convert the captured voltage into digital signal. Here, we only care about the variation of the peak voltage, and the absolute voltage does no matter. So, the voltage drop of the diode does not influence the system performance. Before running the system, the ADC output should be calibrated. We measured the peak voltage by oscilloscope. When the oscilloscope shows a 10 V peak-peak voltage (the received power in the 6.5Ω load is 2 W), the ADC output value is set as $V_{req}$.

### 4.4. Design of the Supply Voltage Adjustment Circuit

The DC-DC converter LT1370 shown in Figure 13b is used as the supplier for the PA. LT1370 can provide 90% efficiency for 12 V 0.5 A output, which is a high value. The output voltage of LT1370 is determined by the voltage level at the feedback pin. A digitally controllable resistor AD5272 is connected to the feedback pin. By sending the command from the FPGA, the dc output voltage of DC-DC converter is adjusted. $V_{s0}$ is the voltage for system working in region B, when system goes into region A, $V_{s}$ increases accordingly. $V_{s}$ is proportional to the dc output voltage. In addition, in our design, $V_{s0}$ is set as 5 V. However in real measurement, this value is adjusted according to the PA loss and Q loss in IMN. The discussion about this adjustment is discussed in next section.

## 5. Measurement Result

### 5.1. Experiment Result for 2 W Output Power

**Figure 14:**
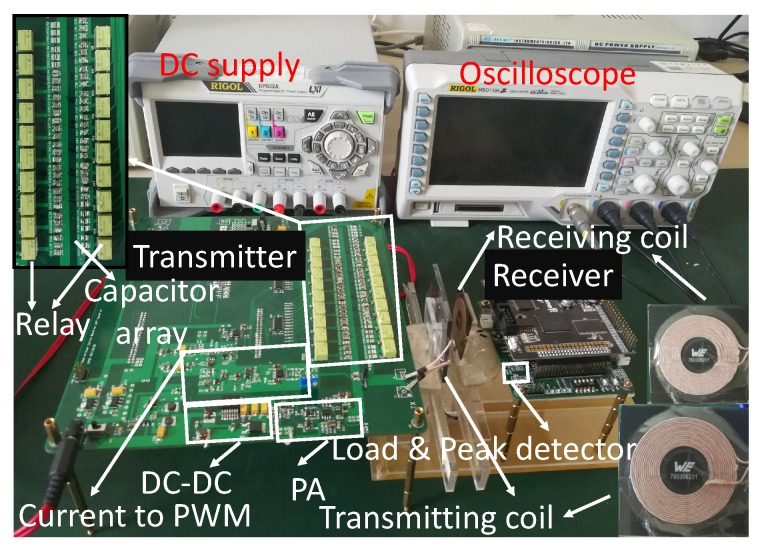
Fabricated adaptive IMN IWPT system and the measurement setup.

The proposed adaptive IMN IWPT system is fabricated, and the measurement setup of the system is shown in Figure 14. In our implementation, EPC2014 GaN PET is employed to implement the class D PA, and EP4CE15F17 FPGA is used as the controller. For the measurement, the input dc voltage was supplied by an external power supply, which is also used to monitor the supply voltage and input current of PA. The power delivered to load is monitored by oscilloscope. The efficiency is calculated from the monitored power delivered to load and the monitored output voltage and current of the power supply. This calculation includes not only the efficiency of the IWPT link, but also the efficiency of the PA and its driver.

The power loss of the control system is considered separately. The control part of the system is composed of relays for capacitor arrays, the current signal detector, the voltage peak detector and the controller FPGA. The relay consumes about 100 mW when it works according to the data sheet. However the existence of the latch makes it consume power only when the relay state need to be changed. Usually, at steady state, the latch could hold the relay state and consumes no power. The current signal detector and the voltage peak detector are made up of separate components, which consume about 34 mW and 50 mW power respectively. The FPGA consumes about 150 mW power, which is the most energy-intensive part in control system. This implementation is just a prototype to verify our algorithm. The power consumption could be further decreased and becomes negligible when the control system is implemented by CMOS integrated circuit.

During the measurement, we compare our adaptive IMN IWPT system with another two IWPT systems: the one uses series resonant capacitors as IMN (Ser-Res IMN), the other one uses fixed IMNs which is designed when *k* = 0.1.

**Figure 15:**
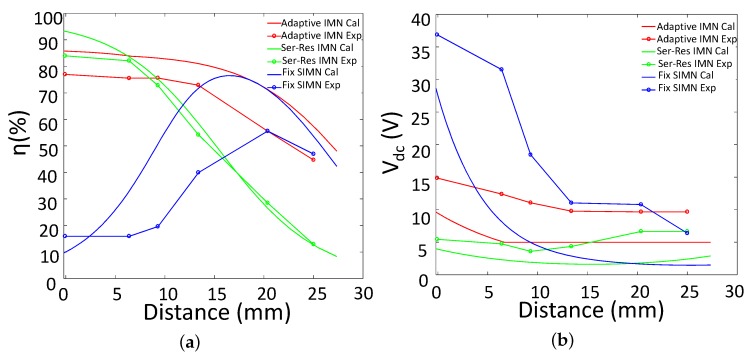
Comparison between simulation and experiment results: (**a**) the efficiency vs. distance; (**b**) the dc supply voltage vs. distance. The power delivered to load is limited to 2 W.

The measured power efficiencies for various distances are presented in Figure 15a. All of the IWPT systems have 2 W output power at the load. The measured value is less than the calculated one, this is because the power loss in PA and IMN capacitors are not considered in calculation.

For the Ser-Res IWPT system, the efficiency is the highest when the distance is smaller than 8 mm. This is because the coils are strongly coupled, the reflected impedance is so large that the loss of the coil resistance can be ignorable. However when the distance increases, the power efficiency decreases sharply, which means series resonant IWPT system is only fit for strongly coupled application. For the fixed IMN IWPT system, the efficiency is only high when the distance is about 20 mm (*k* = 0.1). When the distance changes, the efficiency is decreased significantly, which means that this IWPT system can only adapt to very limited distance variation. This result also agrees with [13].

Compared with Ser-Res IMN IWPT and fixed IMN IWPT, our adaptive IMN IWPT system contains a high power efficiency in the largest range of distance variation. Although the power efficiency of our system is slightly lower than that of Ser-Res IMN IWPT system when distance is smaller than 8 mm, our power efficiency still contains in an acceptable high value (over 76%). Furthermore, our system efficiency keeps a much higher value than Ser-Res IMN IWPT system when the distance varies from 8 mm to 25 mm, which means that our system is more robust for distance variation.

The supply voltage variation of these IWPT systems are presented in Figure 15b. Our design is according to that the $V_{s}$ is 5 V when k<0.4, and increases when k>0.4, which means the dc supply is 4 V when k<0.4 and increases when k>0.4. However, as we indicated above, the power losses of PA and capacitors are introduced in real system, and the power efficiency is smaller than the calculated one. To ensure the constant 2 W output, The dc supply voltage is increased for our adaptive IMN IWPT system. From the plot we can see that when distance is larger than 13 mm, the dc supply is 9 V, and the dc supply increases up to 15 V when the distance approaches 0 mm. This voltage variation is affordable for the DC-DC converter LT1370.

The dc supply for Ser-Res IMN IWPT and fixed IMN IWPT are also adjusted to ensure 2 W power delivered to load. For fixed IMN IWPT system, the dc supply increases sharply when distance approaches 0 mm, this is because the power efficiency is decreased seriously. For Ser-Res IMN IWPT system, the dc supply voltage contains in a low range. This is because when distance is small, the power efficiency is high, when distance increases, although the power efficiency decreases sharply, the reflected resistance is also decreased, which leads to larger PA output power. If the load needed power is much less than 2 W, the dc supply voltage would be much smaller than 5 V, this low dc voltage would also be a challenge for PA design. If the high dc supply is used, the redundant power delivered to load would be wasted. Thus, Ser-Res IMN IWPT is hard to achieve high efficiency for low power application.

### 5.2. Adapt the System to 10 W Output Power

All above discussion is on 2 W output power level, which is a typical requirement for low power devices. In reality, there are also many high power applications with different challenges. Here, we adjust our system to adapt to 10 W output power, which can be used to discuss these challenges.

**Figure 16:**
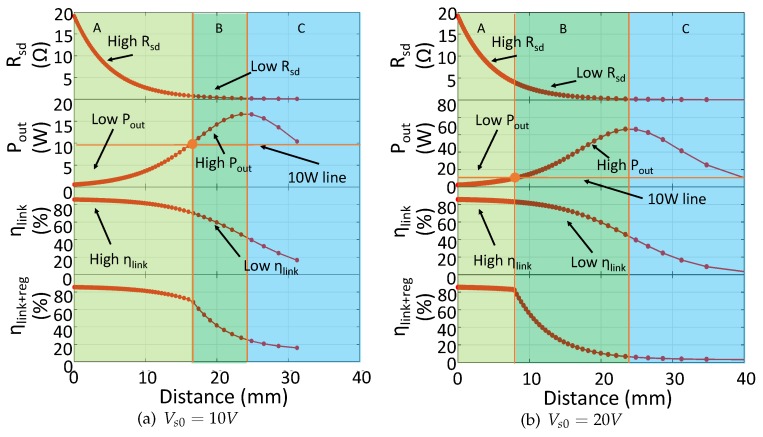
The IWPT system performance when IMN is a series resonant capacitor. The output power is 10 W, the initial supply voltage $V_{s0}$ is 10 V for (**a**) and 20 V for (**b**).

Similar to the analyses in Section 3. We firstly set the SIMN as series resonant, and analysis the system performance under distance variation for 10 W output. Figure 4 is revised as Figure 16, in which the output power level requirement is 10 W, and the initial supply voltage ($V_{s0}$ in algorithm) is 10 V and 20 V.

For the 10 V supply voltage situation, we can see that region A is enlarged and region B is shrunk into a narrow range. This shrink is caused by the increase in load power requirement, which means more power need to be transferred from the source side. For our adaptive IMN IWPT system we use different control method for region A and region B. In region A, the SIMN is simplified to series resonant capacitor, and the supply voltage is increased to meet the output power requirement. In region B, the input impedance of SIMN is adjusted to enhance efficiency and control the output power as well. The shrink of region B means that the opportunity for our system to enhance the system efficiency is decreased. Fortunately, this can be solved by increasing the supply voltage. When the supply voltage is increased into 20 V in Figure 16b, the output power requirement can be accomplished more easier which leads to a larger region B. Of course, increasing supply voltage would increase the DC-DC power loss. In applications, the chosen of suitable supply voltage need to trade off between the DC-DC supply loss level and the SIMN adjustment range. The aim of the trade-off is to ensure a high efficiency in a large distance variation range.

**Figure 17:**
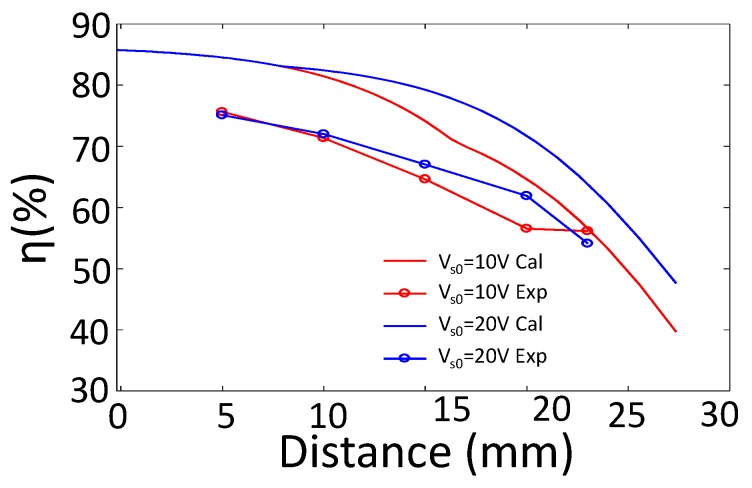
The simulation and experiment results for 10 W output performance.

Figure 17 shows the simulation and experiment results of the modified adaptive IMN IWPT system for 10 W output power. For $V_{s0}=10$ V, region B is about 16 mm to 23 mm, and for $V_{s0}=20$ V, region B is about 9 mm to 23 mm. From 9 mm to 16 mm, Two configurations are in different regions: $V_{s0}=10$ V configuration is in region A, which uses series resonant capacitor as SIMN; $V_{s0}=20$ V configuration is in region B, which uses Γ type SIMN. The experiment result shows that efficiency enhancement for $V_{s0}=20$ V is smaller than the simulation result. This is because Γ SIMN decreases the quality factor, which would increase the harmonic losses of PA.

### 5.3. Comparison with Literatures

Table 3 shows a comparison with the results for previously reported IWPT systems. Because of differences among these systems, such as adjusting method, working frequency, coil geometry, power level, and efficiency calculation, etc., it is hard to compare the system performance directly. We should notice that frequency and the distance are two critical influence parameters, where higher frequency and smaller distance will increase the efficiency. Beside, the purpose of this paper is not to achieve numerical highest efficiency, but to propose a promising adaptive system design. So our comparison below considers both efficiency performance and control strategy.

The systems in [13,24,25] and our system are all focus on controlling IMN to adaptive to coil distance variation. The systems in [13,25] show higher efficiency values than ours. However, the frequencies in these systems are much higher than that in ours and the PA consumptions are not considered. Considering these elements, our system performance is still promising. From the control method point of view, the systems in [13,24,25] are all trying to compensate the mismatch of the imaginary part of the load impedance and retune the system, but the output power level is not controlled. This may lead to power waste if redundant power is transferred to load. The problem of maintaining the load received power stable is solved in our system by monitoring the load voltage and adjusting IMN and $V_{s}$. With our adjustment method, the efficiency could also be enhanced further.

Other than controlling IMN, the adaptive IWPT systems in [18,22] are achieved by controlling load impedance and frequency respectively. When the normalized distance is close, our system performs a higher efficiency. Comparing with [18], our system does not need large frequency bandwidth. In [22], the system focuses on achieving optimal load resistance and maintaining the output power level stable. The optimal load is achieved by adjusting the duty cycle of the regulator, and the output power level is maintained by adjusting supply voltage. Different from this method, we maintain the output power stable by controlling the SIMN input impedance when the coupling is weak and the system is in region B of Figure 4. Because the output is larger than needed and efficiency is small, adjusting supply voltage can only reduce the output power, but has no contribution on efficiency enhancement. However, in our system, the output power is reduced by increasing the SIMN input impedance. With this method, the efficiency can also be increased since the load impedance of PA is increased.

From the comparison, we can see that our system is a promising adaptive IWPT system solution, by which not only the power efficiency can be enhanced, but also the power delivered to load can be controlled.

## 6. Conclusions

In this paper, an adaptive IMN IWPT system is proposed to solve the power efficiency degradation and output power variation when coil distance varies. Two capacitors in SIMN are controlled simultaneously by our control algorithm. As the result, the impedance mismatch is compensated and power delivered to load is adjusted. As far as we know, our system is the first one to achieve a closed loop control solution for IWPT, with which not only the power efficiency can be enhanced, but also the power delivered to load can be adjusted. The experiment results show that our adaptive IMN IWPT system could perform a high power transfer efficiency when the distance varies in a large range from 0 mm to 25 mm (means the coupling coefficient ranges from 0.8 to 0.05). Comparing with the series resonant IWPT system and fixed IMN IWPT system, our adaptive IMN IWPT system achieves up to 31.79% and 60% power transfer efficiency improvement for 2 W output power.

## Figures and Tables

**Table 1:** The parameters for IMN-IWPT system discussed in this paper.

	Coil Tx	Coil Rx
Coil parameters		
Coil order code	760308111	760308201
Outer diameter (mm)	43	28
Turn number	10	15
Inductance (μ H)	6.3	10
Quality factor	80	50
System parameters		
Frequency (kHz)	125	
$V_{s}$ (*V*)	5	
$r_{0}\left(\Omega \right)$	0.1	
$R_{L}\left(\Omega \right)$	6.5	
$P_{req}$ (W)	2	

**Table 2:** Values of the capacitor arrays.

	$C_{0}$	$C_{1}$	$C_{2}$	$C_{3}$	$C_{4}$	$C_{5}$	$C_{6}$	$C_{7}$	$C_{8}$
$C_{m1}$ (nF)	58	1	2	4	8	16	32	64	128
$C_{m2}$ (nF)	-	1	2	4	8	16	32	64	128

**Table 3:** Performance comparison.

	Controlled	Frequency	Diameter	Distance	Normalized	Coupling	Power	Efficiency	Consider
	Component ${}^{1}$	(Hz)	(mm)	(mm)	Distance ${}^{2}$	Coefficient	(W)	(%)	PA/DC-DC ${}^{3}$
[13]	IMN	13.56 M	400/400	0	0	NA	1	85	NO
				400	1	NA	1	80	
				800	2	NA	1	38	
[24]	IMN	13.56 M	40/40	2.5	0.06	0.92	NA	27	PA & DC-DC
				15	0.38	0.22	24 m	49	
				40	1	0.05	NA	10	
[25]	IMN	13.56 M	205/280/280/205 ${}^{4}$	100	0.42	NA	NA	92	NO
[22]	Load	305 k	125/115	55	0.46	0.137	2.7	64	NA
[18]	Frequency	7 M–9.5 M	30/20	10	0.4	NA	174 m	63	NA
Our work	IMN	125 k	43/28	0	0	0.76	2	77	PA
				15	0.35	0.16	2	70	
				25	0.58	0.05	2	45	

${}^{1}$ This column displays the controlled component of the adaptive IWPT system in the literature; ${}^{2}$ The normalized distance is defined as *Normalized* *distance* = *Distance* $/\sqrt{Transmitting\;coil\;diameter*Receiving\;coil\;diameter}$; ^3^ This column indicates whether the efficiency of PA or DC-DC is considered in efficiency calculation; ${}^{4}$ In this literature, four coupling coils are used.

## Abbreviations

The following abbreviations are used in this manuscript:

IWPT	Inductive Wireless Power Transfer
IMN	Impedance Matching Network
SIMN	IMN at Source Side
LIMN	IMN at Load Side
PA	Power Amplifier
Ser-Res IMN IWPT	Series Resonant IMN IWPT
